# Validity and Reproducibility of a Habitual Dietary Fibre Intake Short Food Frequency Questionnaire

**DOI:** 10.3390/nu8090558

**Published:** 2016-09-10

**Authors:** Genelle Healey, Louise Brough, Rinki Murphy, Duncan Hedderley, Chrissie Butts, Jane Coad

**Affiliations:** 1 Massey Institute of Food Science and Technology, School of Food and Nutrition, Massey University, Palmerston North 4442, New Zealand; l.brough@massey.ac.nz (L.B.); j.coad@massey.ac.nz (J.C.); 2 Food, Nutrition and Health, The New Zealand Institute for Plant & Food Research Limited, Palmerston North 4442, New Zealand; duncan.hedderley@plantandfood.co.nz (D.H.); chrissie.butts@plantandfood.co.nz (C.B.); 3 Faculty of Medical and Health Sciences, The University of Auckland, Auckland 1023, New Zealand; r.murphy@auckland.ac.nz

**Keywords:** dietary fibre, short food frequency questionnaire, validation

## Abstract

Low dietary fibre intake has been associated with poorer health outcomes, therefore having the ability to be able to quickly assess an individual’s dietary fibre intake would prove useful in clinical practice and for research purposes. Current dietary assessment methods such as food records and food frequency questionnaires are time-consuming and burdensome, and there are presently no published short dietary fibre intake questionnaires that can quantify an individual’s total habitual dietary fibre intake and classify individuals as low, moderate or high habitual dietary fibre consumers. Therefore, we aimed to develop and validate a habitual dietary fibre intake short food frequency questionnaire (DFI-FFQ) which can quickly and accurately classify individuals based on their habitual dietary fibre intake. In this study the DFI-FFQ was validated against the Monash University comprehensive nutrition assessment questionnaire (CNAQ). Fifty-two healthy, normal weight male (*n* = 17) and female (*n* = 35) participants, aged between 21 and 61 years, completed the DFI-FFQ twice and the CNAQ once. All eligible participants completed the study, however the data from 46% of the participants were excluded from analysis secondary to misreporting. The DFI-FFQ cannot accurately quantify total habitual dietary fibre intakes, however, it is a quick, valid and reproducible tool in classifying individuals based on their habitual dietary fibre intakes.

## 1. Introduction

Dietary fibre are non-digestible plant polysaccharides found in high amounts in fruits, vegetables, breads and cereals, legumes and nuts and seeds. Dietary fibre has been shown to have important implications on human health, including preventing and alleviating constipation, reducing gastrointestinal cancer incidence and blood glucose levels, lowering blood cholesterol levels and blood pressure, and beneficially modulating gut microbiota [1]. It is also possible that the efficacy of a dietary intervention is altered as a result of the influence habitual dietary fibre intake has on gut microbiota responsiveness and host outcomes. Therefore, being able to quickly assess an individual’s habitual dietary fibre intake and classify individuals based on their dietary fibre intakes will prove useful in clinical practice and in nutrition and health research. Dietary assessment methods such as diet records and food frequency questionnaires have inherent limitations such as being difficult to complete accurately, time-consuming and may not accurately assess a person’s habitual diet [2]. A small number of dietary fibre assessment questionnaires have been developed, however these questionnaires assess general dietary behaviours, do not estimate total dietary fibre amounts, and/or do not classify individuals based on habitual dietary fibre intakes [3,4,5,6]. Therefore, the primary aim of this study was to determine whether a newly developed dietary fibre intake short food frequency questionnaire (DFI-FFQ) can accurately classify individuals based on their habitual dietary fibre intake and the secondary aim of the study was to determine whether the DFI-FFQ can accurately quantify total habitual dietary fibre intakes.

## 2. Methods

### 2.1. Subjects

Participants were recruited via email and poster advertisement in multiple locations around Palmerston North, New Zealand. A diverse cross-section of the population was targeted to help ensure a good representation of the New Zealand population was recruited. Sixty-eight individuals provided informed consent to participate in this study, of which fifty-two healthy participants met the inclusion criteria (aged >19 and <65 years, healthy, BMI >18.5 and <30 kg/m^2^, no significant weight loss or weight gain within the past year, no significant dietary change within the past year, not pregnant or breastfeeding, no food intolerances which cause gastrointestinal symptoms (i.e., lactose intolerance, gluten sensitivity), no adverse gastrointestinal symptoms, non-smoker and not high alcohol consumers). Participants completed the DFI-FFQ twice, at least 2 weeks apart, and the comprehensive nutrition assessment questionnaire (CNAQ) once. The DFI-FFQ was completed initially, followed by the CNAQ, and lastly the repeated DFI-FFQ was completed. The CNAQ and DFI-FFQ were both completed online. An energy intake: basal metabolic rate (EI:BMR) of <1.1 and >2.19 was used to exclude participants who appeared to have over- or under-reported using the CNAQ [7]. Ethical approval was obtained from the Massey University Human Ethics Committee (Southern A, Application 15/34).

### 2.2. Development of the DFI-FFQ

The DFI-FFQ (Figure S1) was designed to quickly and accurately classify individuals as low, moderate or high habitual dietary fibre consumers and quantify an individual’s habitual dietary fibre intake (g/day). The DFI-FFQ consists of five high dietary fibre containing food groups (vegetables, fruits, breads and cereals, nuts and seeds and legumes) which account for 73.5% of the dietary fibre in a typical New Zealand diet [8]. Examples of what one serve is equivalent to, for each food group, is detailed within the DFI-FFQ. The frequency of consumption for the average number of serves consumed over the past year, was given as follows: Never, <1/month, 1–3/month, 1/week, 2–4/week, 5–6/week, 1/day, 2/day, 3/day, 4/day, 5/day and 6+/day.

### 2.3. DFI-FFQ Scoring Sheet

A scoring sheet was developed to quantify the amount of dietary fibre consumed and to classify individuals as low, moderate and high dietary fibre consumers. FoodWorks version 7.0.3016 (Xyris Software Pty Ltd., Brisbane, Queensland, Australia) was used to quantify the average amount of dietary fibre provided by the five food groups for each frequency of consumption. An individual’s total dietary fibre intake was calculated by adding together the average amount of dietary fibre consumed from each food group in relation to the number of serves consumed.

### 2.4. Dietary Fibre Classification

The cut-offs used to classify individuals based on their dietary fibre intakes are outlined in Table 1. The high dietary fibre intake cut-offs were selected to reflect the New Zealand Ministry of Health recommended dietary fibre intake guidelines; >25 g/day for females and >30 g/day for males [9]. The low dietary fibre intake cut-offs were selected as the median dietary fibre intake in New Zealand was 17.5 g/day for females and 22.1 g/day for males, which are below recommended amounts [8]. Similar cut-offs have been used previously however the specific cut-offs used in this study were modified to be applicable to a New Zealand population [3].

### 2.5. Dietary Assessment Method Used for Comparison

The Monash University online CNAQ was used for comparison with the DFI-FFQ. The 297-item food frequency questionnaire has been shown to be valid in assessing habitual dietary intakes when compared to four 7-day food records, each completed three months apart [10].

### 2.6. Statistical Analysis

We aimed to recruit enough participants to ensure that correlations over 0.7 would be statistically significant and that the assumptions of *chi-squared* tests would not be over stretched. The relationship between results of the DFI-FFQ when compared to the CNAQ was determined using Spearman correlation, Pearson correlation, Bland-Altman plot, *chi-squared* test and linear weighted kappa score. Test-retest repeatability was assessed using Pearson correlation, Bland-Altman plot and Cronbach’s alpha. *T*-tests were used to determine whether there were any differences in dietary fibre intakes between the DFI-FFQ and CNAQ and the repeated DFI-FFQ. A *p* value of < 0.05 is considered significant. Statistical analysis was carried out using GenStat 17th edition (VSNi Ltd., Hemel Hempstead, UK), Minitab 16th edition (Cronbach’s alpha) (Minitab Inc., State College, PA, USA) and the calculator at http://vassarstats.net/kappa.html (kappa score) [11].

## 3. Results

All eligible participants (*n* = 52) completed the study. The data from 28 participants (54%) were used as the data from 24 participants (46%) were excluded from the analysis secondary to likely misreporting on the CNAQ; with 18 participants (34.5%) having over-reported and six participants (11.5%) having under-reported their energy intakes. The group mean EI:BMR was 2.8 (SD 4.7) prior to exclusion and reduced to 1.6 (SD 0.3) after exclusion. Participant characteristics, total dietary fibre intakes and classifications determined by the DFI-FFQ and CNAQ are summarised in Table 2. The median dietary fibre intake in New Zealand (20.3 g/day) [8] is similar to the average dietary fibre intake of the study cohort, with dietary fibre intakes from both groups being below the New Zealand recommended dietary fibre intake guidelines [9]. The DFI-FFQ took on average 3.5 min to complete in comparison to the estimated completion time of 20–40 min for the CNAQ.

When comparing the DFI-FFQ to the CNAQ for dietary fibre classification, exact agreement occurred 79% of the time and gross misclassification occurred 7% of the time (Table 3). There was a significant difference in dietary fibre intakes between the DFI-FFQ and CNAQ (CNAQ was on average 5 g/day higher than the DFI-FFQ). The two dietary assessment methods were however correlated (Pearson correlation 0.65, Spearman correlation 0.53). A *chi-squared* test indicated an association between the classifications based on the DFI-FFQ and CNAQ (*p* = 0.002) and the linear weighted kappa score showed good agreement [12] (Table 4). The Bland-Altman plot is available within the Supplementary information (Figure S2A).

Pearson correlation (0.94) and Cronbach’s alpha (0.97) showed that the repeated DFI-FFQ correlated. The estimated dietary fibre intake from the second DFI-FFQ was significantly lower than the first DFI-FFQ by 1.8 g/day (Table 4). The Bland-Altman plot is available within the Supplementary information (Figure S2B).

## 4. Discussion

Presently, there are no known short dietary fibre intake questionnaires that are able to classify individuals based on their habitual dietary fibre intake. Having the ability to be able to quickly and accurately classify an individual based on their dietary fibre intake will prove useful as low dietary fibre intakes have been associated with poorer health outcomes [13]. This study has shown that the DFI-FFQ can accurately classifying individuals based on their habitual dietary fibre intakes.

There was however, a significant difference in habitual dietary fibre intakes between the repeated DFI-FFQs and the DFI-FFQ and CNAQ, which suggests the DFI-FFQ might not accurately quantify total habitual dietary fibre intakes. Research has shown that large food item FFQs overestimate fruit and vegetable consumption, which may help explain the higher dietary fibre intakes determined from the CNAQ [14]. The addition of other dietary fibre contributing food groups, such as cakes and muffins, pies and pastries and biscuits, to the DFI-FFQ may have helped to improve the questionnaire’s accuracy in quantifying total habitual dietary fibre intakes as these food groups collectively contribute 6.3% of the dietary fibre in a typical New Zealand diet [8]. Another reason why the DFI-FFQ may not have been able to accurately quantify total habitual dietary fibre intakes may be related to the serving size examples provided. The examples provided did not include all possible foods within a particular food group and relied on participants to use their own judgement regarding the number of serves consumed for foods that were not specifically listed.

There are a handful of short questionnaires that have been developed to assess dietary fibre intakes however these questionnaires assess general dietary behaviours [4,5,6], do not estimate total dietary fibre amounts [4,5,6], and/or do not classify individuals based on habitual dietary fibre intakes [3,4,5,6]. The DFI-FFQ is novel as it can accurately classify individuals based on habitual dietary fibre intake. Unlike previously developed questionnaires, the DFI-FFQ was validated against an FFQ which assesses dietary intake over the past year, providing a more accurate account of long term rather than current dietary fibre intakes. Additionally, some of the questionnaires were validated using fairly homogenous populations, such as factory workers [3] and patients [5], making these questionnaires less useful in more diverse populations, such as in this study.

When comparing the study cohorts average dietary fibre intake to the Adult Nutrition Survey data [8] it appeared the study cohort has a similar dietary fibre intake to the New Zealand population. Therefore, the DFI-FFQ is a valid tool for classifying individuals based on their habitual dietary fibre intakes in New Zealand. In countries where dietary fibre intakes are distinctly different from New Zealand, the DFI-FFQ may need to be re-validated in these populations.

Forty-six percent of participants were excluded from the study secondary to misreporting on the CNAQ, which reduced the data available for analysis. A known limitation of FFQs is the high rate of misreporting, however the rate of misreporting in this study was much higher than previously reported [15]. It may therefore be useful to compare the DFI-FFQ to another dietary assessment method (i.e., 3- or 7-day diet records, or shorter validated FFQ) to confirm these results. The sample size for this study was small however a sufficient number of participants were recruited based on the sample size calculations, even after exclusion for misreporting. Additionally, other dietary questionnaire validation studies have similarly small participant numbers [16,17]. Despite the limitations discussed, we believe the DFI-FFQ will be a valuable tool in research and clinical practice as it is quick to complete (3.5 min on average), has low respondent burden and is a valid and reproducible method of classifying individuals based on their habitual dietary fibre intakes.

## 5. Conclusions

The DFI-FFQ has been shown to be a quick, valid and reproducible tool in classifying individuals based on their habitual dietary fibre intakes. The DFI-FFQ cannot however, accurately estimate total habitual dietary fibre intakes.

## Figures and Tables

**Table 1:** The dietary fibre intake cut-offs used to classify individuals as low, moderate and high dietary fibre consumers.

	Females	Males
Low	<18 g/day	<22 g/day
Moderate	18–24.9 g/day	22–29.9 g/day
High	≥25 g/day	≥30 g/day

**Table 2:** Characteristics, dietary fibre intakes and classifications for the study participants.

Mean (SD)	Male (*n* = 8)	Female (*n* = 20)	Total (*n* = 28)
**Participant characteristics**			
Age (years)	40 (11.02)	38 (9.37)	39 (9.91)
BMI (kg/m^2^)	24 (1.9)	23 (3.1)	24 (2.82)
Ethnicity (No.)			
New Zealand European	4	14	18
Asian	3	0	3
Maori	0	2	2
Other	1	4	5
**Dietary fibre intakes and classifications**			
***DFI-FFQ***			
Dietary fibre intake (g/day)	27 (11.77)	23 (10.33)	24 (10.85)
Dietary fibre classification (No.)			
Low	2	5	7
Moderate	2	4	6
High	4	11	15
***Monash CNAQ***			
Dietary fibre intake (g/day)	31 (11.35)	29 (9.43)	29 (10.09)
Dietary fibre classification (No.)			
Low	1	4	5
Moderate	3	1	4
High	4	15	19

DFI-FFQ: dietary fibre intake short food frequency questionnaire; CNAQ: comprehensive nutrition assessment questionnaire; SD: standard deviation.

**Table 3:** Comparison in dietary fibre classification between the comprehensive nutrition assessment questionnaire (CNAQ) and the dietary fibre intake food frequency questionnaire (DFI-FFQ).

		CNAQ			Total
		Low	Moderate	High	
DFI-FFQ	Low	5 (18%)	0 (0%)	2 (7%)	7 (25%)
	Moderate	0 (0%)	3 (11%)	3 (11%)	6 (21%)
	High	0 (0%)	1 (3%)	14 (50%)	15 (54%)
Total		5 (18%)	4 (14%)	19 (68%)	28 (100%)

**Table 4:** Correlation and test-retest repeatability statistical analysis.

**Correlation between DFI-FFQ and CNAQ**		***p* Value**
Pearson correlation	0.65	<0.001
Spearman correlation	0.53	0.001
*Chi-square* test	9.6	0.002
Linear weighted kappa *	0.68	
Standard error	0.14	
Magnitude of agreement	Good	
Bland-Altman plot		
Limits of agreement (g/day)	−12.5–22.6	
Standard error	1.7	
Mean difference (g/day)	5	0.007
**Test-Retest Repeatability**		***p* value**
Pearson correlation	0.94	<0.001
Cronbach’s alpha	0.97	
Bland-Altman plot		
Limits of agreement (g/day)	−6.0–9.6	
Standard error	0.72	
Mean difference (g/day)	1.8	0.019

CNAQ: comprehensive nutrition assessment questionnaire; DFI-FFQ: dietary fibre intake short food frequency questionnaire; * One category disagreement had a weight of ¾.

## Supplementary Materials

Supplementary File 1
