# Supplementary material for: Validity and Reproducibility of a Habitual Dietary Fibre Intake Short Food Frequency Questionnaire
Source: Nutrients. 2016 Sep 10;8(9):558. doi: 10.3390/nu8090558 (PMC5037543; doi:10.3390/nu8090558)
Supplement: Supplementary File 1 [file nutrients-08-00558-s001.docx]

**Supplementary Materials: Validity and Reproducibility of a Habitual Dietary Fibre Intake Short Food Frequency Questionnaire**

Genelle Healey, Louise Brough, Rinki Murphy, Duncan Hedderley, Chrissie Butts and Jane Coad


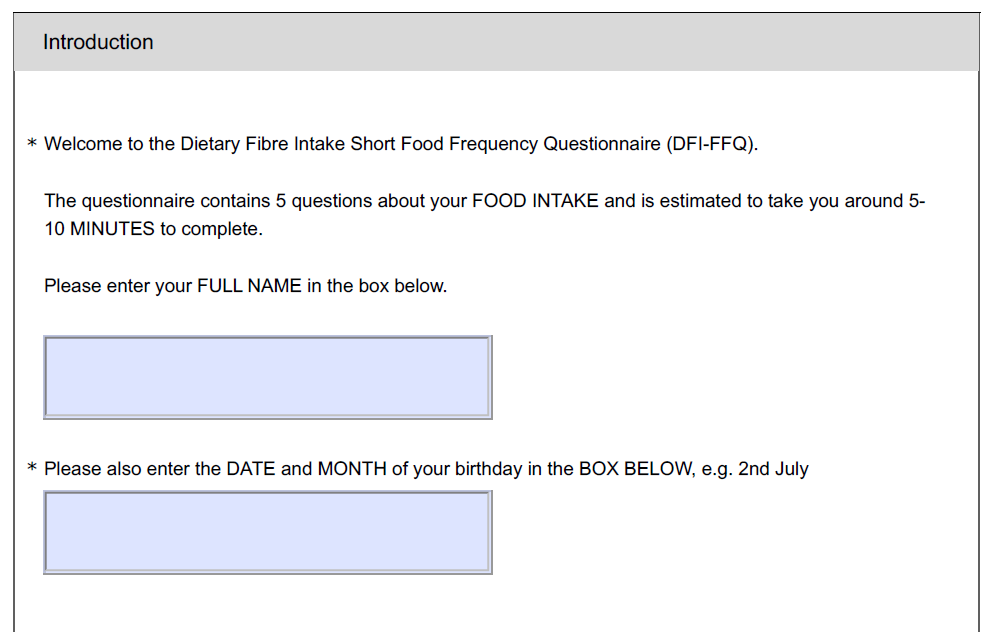


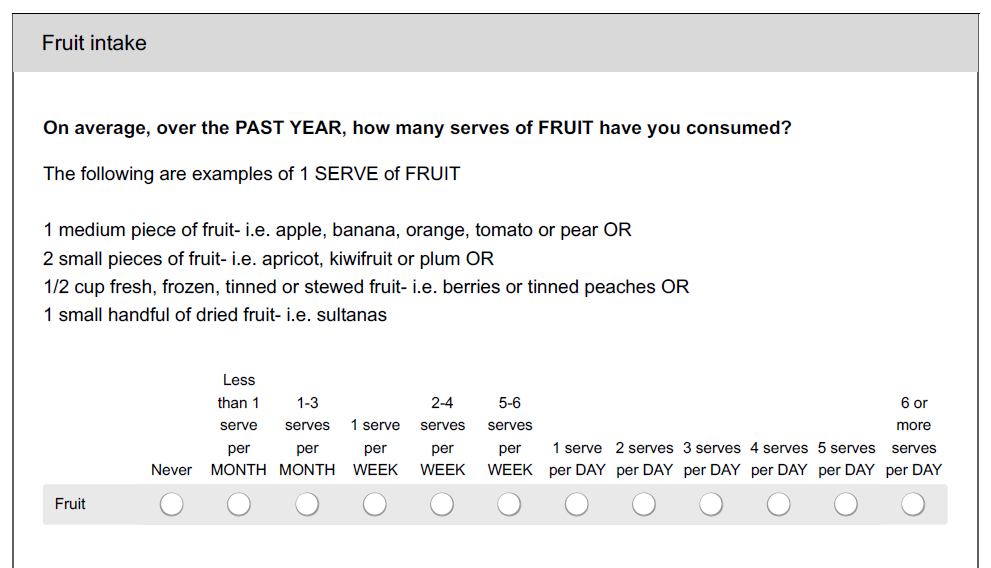


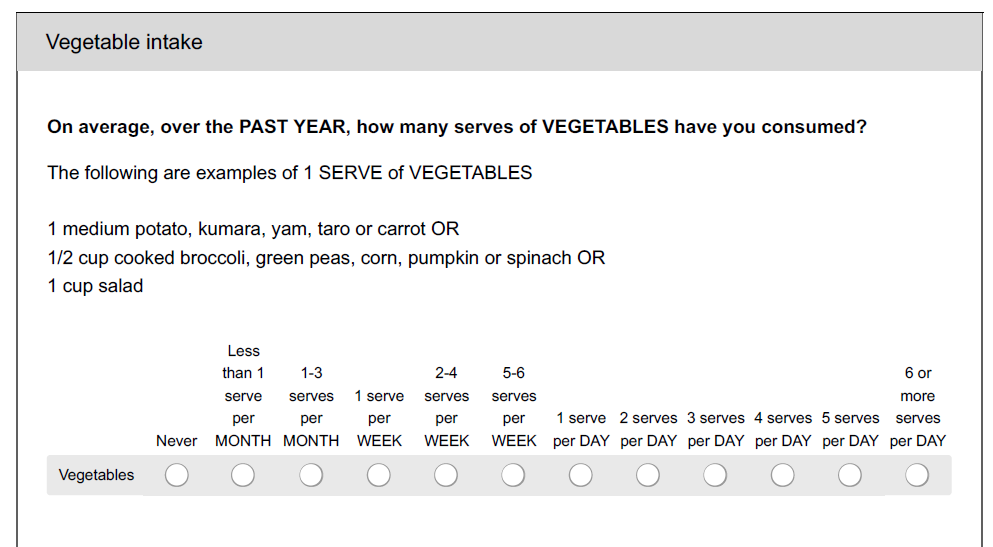


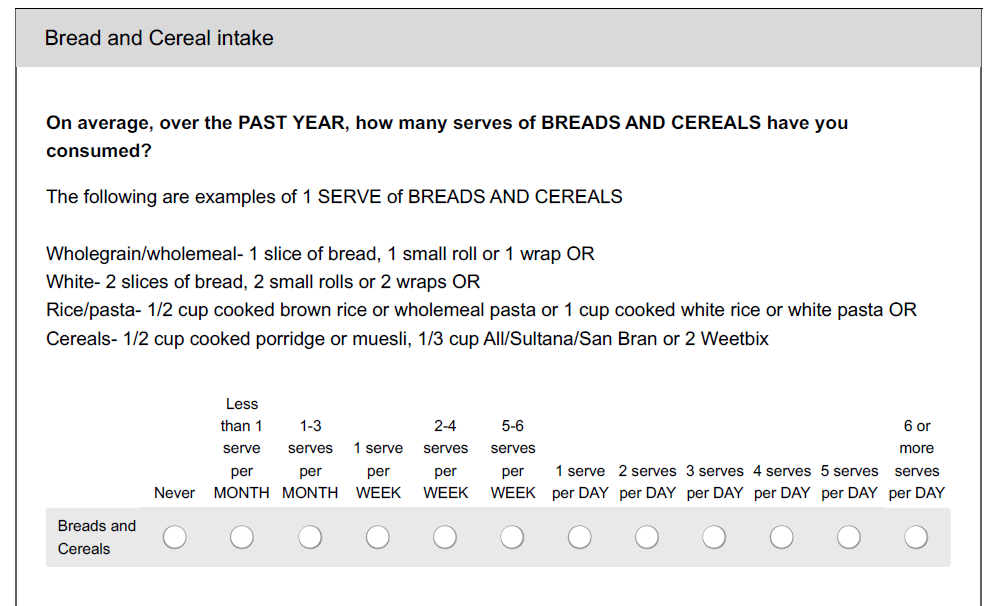


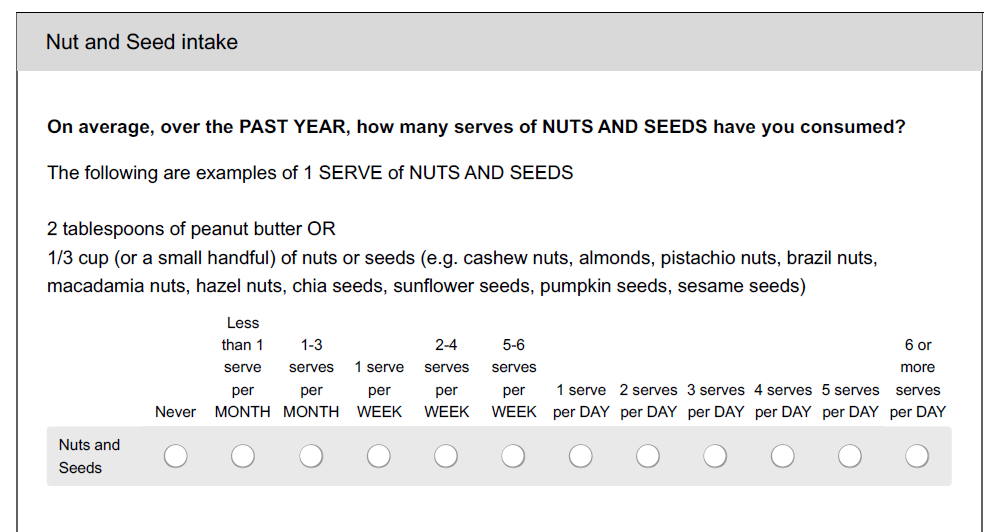


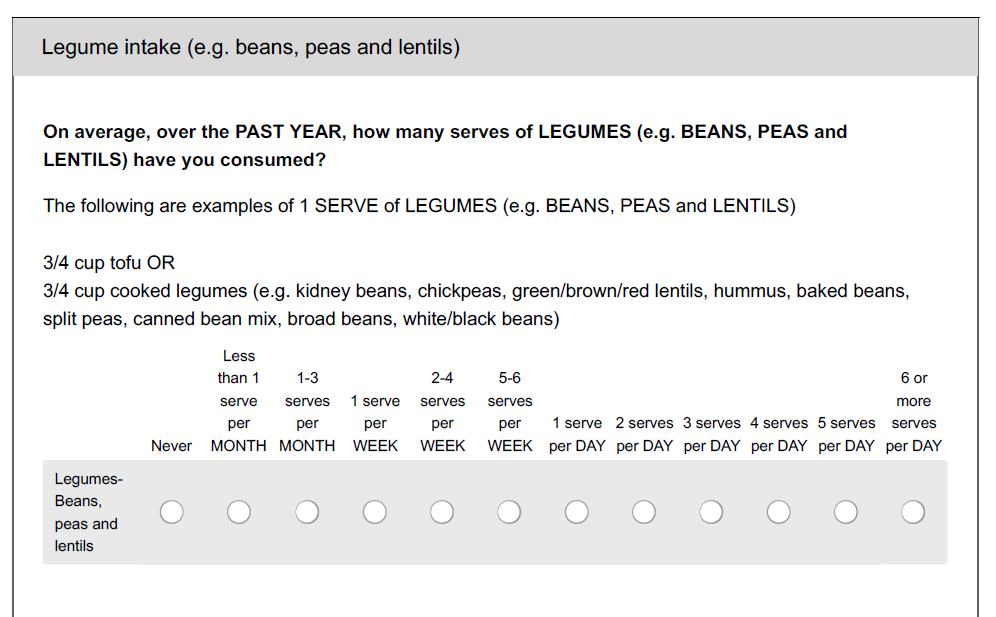


**Figure S1.** Example of the habitual dietary fibre intake short food frequency questionnaire which was generated using SurveyMonkey.

| 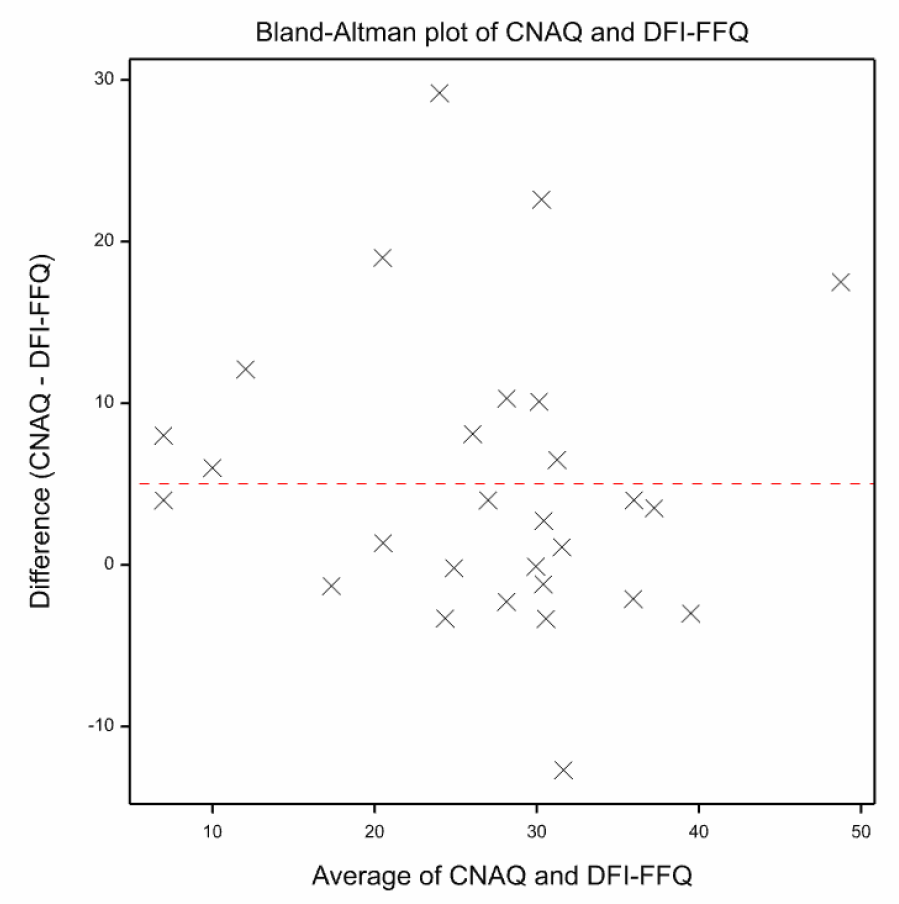  (**A**) |
| --- |
| 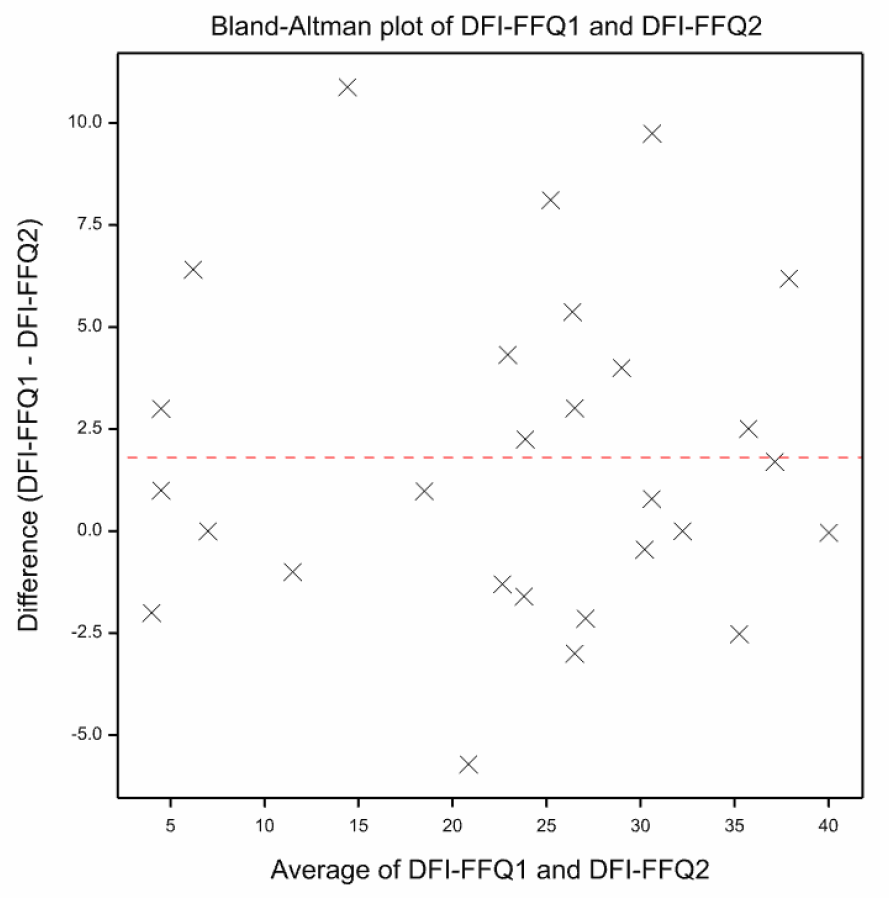  (**B**) |

**Figure S2.** Bland-Altman plots for the comparison between the CNAQ and DFI-FFQ (**A**) and the repeated DFI-FFQs (**B**).
